# Floral Resource Integration: Enhancing Biocontrol of *Tuta absoluta* Within Sustainable IPM Frameworks

**DOI:** 10.3390/plants14030319

**Published:** 2025-01-22

**Authors:** Moazam Hyder, Inzamam Ul Haq, Muhammad Younas, Muhammad Adeel Ghafar, Muhammad Rehan Akhtar, Zubair Ahmed, Aslam Bukero, Youming Hou

**Affiliations:** 1 State Key Laboratory of Agricultural and Forestry Biosecurity, Key Laboratory of Biopesticides and Chemical Biology, MOE, College of Plant Protection, Fujian Agriculture and Forestry University, Fuzhou 350002, China; sahito.2k10pt192@hotmail.com (M.H.);; 2 Vector-Borne Virus Research Center, State Key Laboratory of Ecological Pest Control for Fujian and Taiwan Crops, Fujian Agriculture and Forestry University, Fuzhou 350002, China; 3 Department of Entomology, Sindh Agriculture University, Tandojam 70050, Pakistan

**Keywords:** biological control agents, floral resource management, integrated pest management, sustainable agriculture, *T. absoluta*

## Abstract

The tomato leaf miner, *Tuta absoluta*, is a pest threatening global tomato production. This pest’s adaptability and resistance to chemical insecticides have necessitated integrated pest management (IPM) strategies prioritizing sustainable alternatives. This review explores the role of biological control agents (BCAs) in managing *T. absoluta* populations, emphasizing the integration of floral resources to enhance their efficacy. Predatory mirids such as *Macrolophus pygmaeus* and *Nesidiocoris tenuis* and parasitoids such as *N. artynes* and *Trichogramma* spp. are pivotal in pest suppression; however, their performance depends on nutritional and habitat support. Floral resources provide essential sugars and proteins, improving the longevity, fecundity, and predation efficiency of these BCAs. This review synthesizes case studies highlighting the benefits of selected flowering plants, such as *Lobularia maritima* and *Fagopyrum esculentum*, in supporting predator and parasitoid populations while minimizing advantages to *T. absoluta*. Mechanisms such as nectar quality, floral accessibility, and spatial–temporal resource availability are explored in detail. Additionally, the challenges of selective floral attraction, microbial impacts on nectar composition, and the unintended support of non-target organisms are discussed. This review proposes targeted floral management strategies to optimize BCA performance within IPM systems by integrating ecological and chemical insights. This approach offers a pathway toward reducing chemical pesticide reliance, fostering sustainable agriculture, and mitigating the economic impacts of *T. absoluta* infestations.

## 1. Introduction

The tomato leaf miner, *Tuta absoluta* Meyrick (Lepidoptera: Gelechiidae), is a highly destructive pest significantly threatening tomato *Solanum lycopersicum* L. (Solanales: Solanaceae) cultivation. Originating from South America, this pest has spread globally, becoming a primary agricultural concern. Since its introduction to Europe—initially recorded in eastern Spain in 2006—it has proliferated across several European, Middle Eastern, and North African countries, inflicting substantial yield losses and economic damage [1]. In some cases, *T. absoluta* has caused yield reductions of up to 100% in severely infested fields, with reported losses of up to 1.2 billion USD annually in affected regions, including parts of South America, Europe, and the Middle East [1,2]. The adaptability of *T. absoluta* to various climates, combined with its high reproductive rate, has facilitated its spread from its native range in South America to several regions, including Europe, the Middle East, and parts of Asia and Africa, where it continues to escalate pressure on tomato production systems [1,3]. To counter this pest, the most commonly adopted management approach has involved chemical insecticides, often applied according to a calendar-based schedule in large-scale open-field tomato systems, especially those catering to processing industries [4]. While this approach can effectively reduce pest populations, the overreliance on chemical controls presents significant challenges, notably in regions such as South America, where repeated applications have led to increased resistance among *T. absoluta* populations [5,6]. This resistance spans various chemical classes, including pyrethroids and organophosphates, complicating pest management and increasing costs and health risks. In addition, the indiscriminate use of pesticides has negative consequences for non-target species, particularly natural enemies such as predatory insects and parasitoids, which are critical for sustainable pest management [4].

Biological control offers a promising alternative to chemical management, using natural enemies such as predatory mirid bugs, *Macrolophus pygmaeus* (Rambur) (Hemiptera: Miridae) and *Nesidiocoris tenuis* Reuter (Hemiptera: Miridae), which are widely used in both greenhouse and open-field settings [7,8,9]. These predators effectively control *T. absoluta* by preying on its eggs, although they perform less efficiently on larvae [10,11]. To supplement these predators, larval parasitoids can be highly beneficial. In Mediterranean regions, parasitoid species such as *Necremnus artynes* Walker, *Stenomesiu japonicus* Ashmed (Hymenoptera: Eulophidae), and *Bracon nigricans* Szépligeti (Hymenoptera: Braconidae) have been successfully used to target *T. absoluta* larvae [12,13,14].

Recent studies indicate that the effectiveness of these biological agents can be further enhanced through floral resources. Parasitoids, for example, rely on sugar-rich diets to maximize their reproductive potential and lifespan. Nectar from flowers has been shown to support parasitoids by increasing their longevity and abundance, thereby strengthening their biological control services [15,16,17]. Studies have specifically highlighted the potential of nectar to boost the fitness of *T. absoluta* parasitoids, such as *N. artynes*, which live longer when feeding on floral nectar in the absence of the pest [18]. Additionally, specific parasitoids such as *N. tutae*, *S. japonicus*, and *B. nigricans* require a protein-rich diet to sustain egg production, often obtained by feeding on *T. absoluta* larvae [19,20]. While these findings support the role of floral resources in enhancing the performance of biocontrol agents, the specific mechanisms by which flowers contribute to improved pest control of *T. absoluta* remain poorly understood. Although the presence of flowers has been associated with enhanced fitness and longevity of certain biocontrol agents, such as predators and parasitoids, the exact ways in which floral traits—such as nectar quality, accessibility, and nutrient composition—translate into better pest control outcomes need further exploration. The aim of this review is to examine these mechanisms of floral resource utilization in the biocontrol of *T. absoluta*, providing a detailed analysis of how different floral attributes might optimize the performance of predators and parasitoids against this pest. This review will synthesize current research on the interactions between floral resources and biocontrol agents, ultimately contributing to a more targeted and effective IPM strategy for *T. absoluta*.

## 2. Biological Control Agents and Habitat Enhancements for Effective *T. absoluta* Management

Biological control strategies for managing *T. absoluta* primarily involve natural predators and parasitoids to reduce pest populations sustainably [21]. These agents play a key role in IPM systems, offering a chemical-free alternative for pest control. The effectiveness of biological control depends on ecological factors such as the availability of floral resources, habitats, and environmental stability, which provide sustenance and shelter for biocontrol agents [22]. In the Mediterranean basin, where *T. absoluta* first invaded, conservation biological control has been particularly successful, primarily utilizing the mirid predators *M. pygmaeus* and *N. tenuis* [23]. These generalist predators, which feed on various pests, are most effective against *T. absoluta* eggs; however, their impact on larvae is limited, with less than one larva consumed daily from the second instar onward [24]. To enhance control, studies suggest combining mirid predators with other natural enemies, such as hymenopteran parasitoids, to target various pest stages [25]. Additionally, habitat improvements—such as introducing flowering plants or non-crop refuges—can support biocontrol agents by providing nectar and shelter, thereby boosting their population and enhancing their effectiveness in pest control. This section explores key biocontrol agents for *T. absoluta* control and highlights the ecological conditions, particularly the role of floral resources and habitat enhancements that contribute to their effectiveness.

### 2.1. Key Biocontrol Agents for T. absoluta

Biological control has emerged as a vital alternative for managing *T. absoluta*, given the challenges posed by chemical controls, including issues such as insecticide resistance; however, identifying effective natural enemies for a newly invasive pest is challenging, as *T. absoluta* is associated with many predators and parasitoids [26]. Currently, only four key species have been commercialized and widely used for *T. absoluta* control: the egg parasitoids *Trichogramma pretiosum* and *Trichogramma achaeae* (Hymenoptera: Trichogrammatidae) and the predatory mirids *N. tenuis* and *M. pygmaeus* [3,12]. While these agents were initially identified fortuitously or based on ease of mass rearing, they have since become foundational in *T. absoluta* management. *N. tenuis*, for example, was observed preying on *T. absoluta* in Spanish tomato crops shortly after the pest’s detection there [24]. This predator effectively controls *T. absoluta* by targeting its eggs and early larval stages, thereby helping reduce pest populations before larvae can cause significant damage to plants; however, *N. tenuis*, while effective as a biocontrol agent, can cause visible and sometimes economically significant plant damage due to phytophagy under low prey levels [27,28,29], prompting suggestions to exploit genetic variation to mitigate these effects [30].

*M. pygmaeus*, another major predator, is widely used in greenhouse tomato crops. It effectively suppresses *T. absoluta* by feeding on eggs and young larvae. Dissimilar to *N. tenuis*, *M. pygmaeus* is less likely to harm plants. Still, it does require alternative food sources, such as pollen or additional prey, to sustain its population and support reproduction [7]. This dependency limits its efficacy in open fields where food sources may be scarce, especially if *T. absoluta* populations are low. Both predatory mirids are synovigenic, meaning their reproductive success depends on continued food intake during adulthood. Studies suggest that when floral resources are available, these predators can increase their longevity and effectiveness in pest control [31].

The egg parasitoids *T. pretiosum* and *T. achaeae* have also been utilized, along with other species such as *N. artynes* and *B. nigricans*, which are important for targeting the larval stages of *T. absoluta,* though typically on a smaller scale and in controlled environments such as greenhouses. These parasitoids lay eggs within *T. absoluta* eggs, thereby preventing larval emergence. Because they target the pest at its earliest stage, *Trichogramma* species are valuable in reducing pest populations before significant damage occurs; however, their efficacy is constrained by the need for frequent and high-volume releases to maintain control, especially in larger fields [32,33]. The relatively short lifespans of *Trichogramma* species necessitate consistent reintroduction in the absence of stable populations, primarily limiting their application to greenhouses. Their effectiveness relies heavily on the rapid reproduction cycle and the ability to target multiple generations of *T. absoluta*. Still, this requirement for frequent release poses economic challenges in large-scale open-field settings [34].

The parasitoid *N. artynes*, an idiobiont ectoparasitoid that attacks *T. absoluta* larvae, has shown promise in pest control, particularly in the Mediterranean region. Native to the Palearctic, *N. artynes* primarily targets larvae of *T. absoluta* and has adapted well to Mediterranean climates where the pest is particularly damaging [35,36]. This parasitoid attacks second and third larval instars, feeding destructively on the pest’s body tissues. Synovigenic in nature, *N. artynes* females emerge with a limited number of mature eggs and require steady food sources to sustain oogenesis and maximize reproductive output [18,33]. Access to floral nectar or honeydew significantly boosts the reproductive success and longevity of *N. artynes*, enhancing its impact on *T. absoluta* populations. Research indicates that feeding on floral resources prolongs adult lifespans, which can increase pest suppression by sustaining *N. artynes* populations during periods of low pest density. Despite its adaptability and larval targeting capabilities, further research is necessary to optimize its commercial use in *T. absoluta* management programs [18].

Biological control agents such as *N. tenuis* and *M. pygmaeus* effectively suppress *T. absoluta* by targeting its eggs and larvae, the most vulnerable stages of the pest’s lifecycle. These predators exhibit a flexible feeding behavior, relying on alternative prey and plant-derived resources, such as floral nectar and pollen, to sustain their populations. As noted by Biondi et al. [3], access to alternative prey and supplementary food sources significantly enhances these species’ survival, fecundity, and predation efficiency. Similarly, *Trichogramma* parasitoids focus on egg parasitism, preventing larval emergence and mitigating foliar damage, with frequent releases enhancing their utility in greenhouse settings. *N. artynes*, an ectoparasitoid targeting larvae, disrupts the pest’s lifecycle and reduces crop damage, with its host-feeding behavior further boosting effectiveness when supported by floral nectar. Collectively, these biocontrol agents provide a comprehensive and sustainable approach to *T. absoluta* management, promoting pest control in tomato cultivation [23].

### 2.2. Role of Supplemental Floral Resources in Supporting Biocontrol Agent Performance Needs

The availability of floral resources, such as nectar and pollen, is crucial in enhancing the fitness, longevity, and reproductive success of biocontrol agents targeting *T. absoluta*. Floral nectar, rich in sugars such as sucrose, glucose, and fructose, is an essential energy source for parasitoids and predators [37]. Several studies have highlighted the positive effects of flowering plants, such as *Lobularia maritima* (Brassicales: Brassicaceae) and *Fagopyrum esculentum* (Caryophyllales: Polygonaceae), on the performance of natural enemies. For example, nectar from *L. maritima* has increased the longevity and fecundity of parasitoids such as *N. artynes* and predators such as *M. pygmaeus* [18,19,38]. Furthermore, recent research by [39] demonstrated that *L. maritima* flowers significantly improve the fitness of *D. gelechiidivoris* and enhance the biological control of *T. absoluta* by parasitoids. These findings underscore the vital role of nectar sources, such as those provided by *L. maritima*, in supporting the efficacy of biocontrol agents in tomato agroecosystems. In a field study conducted in 2019, eulophids, including *N. tutae* and braconids, were observed on *L. maritima* flower strips adjacent to tomato fields, further illustrating the importance of supplemental floral resources in improving biocontrol outcomes.

Pollen is a protein source, enhancing reproduction, especially for synovigenic species that rely on adult-acquired resources to produce eggs. Flowering plants around or within tomato fields—such as *Verbena* × *hybrid* Voss (Lamiales: Verbenaceae) and *Scaevola aemula* (Asterales: Goodeniaceae) can provide essential nutrition that supports the survival and reproduction of key predators such as *M. pygmaeus* and *N. tenuis*. For example, flowers such as *Calendula officinalis* (*Asteraceae*) planted in the margins of tomato crops have successfully been used to maintain populations of *M. pygmaeus*, resulting in earlier colonization and increased population densities in tomato crops [40,41]. This integration of flowering plants is essential in regions such as Northeast Spain and Southeast France, where predator population stability is crucial for pest suppression [42,43]. To improve the biocontrol of *T. absoluta*, Table 1 at the end summarizes key floral species such as *L. maritima* and *F. esculentum*, detailing their benefits in enhancing the longevity, reproduction, and foraging of biocontrol agents within IPM strategies. Adding such floral resources not only supports the nutritional needs of these mirid predators but also contributes to their persistence and dispersal, especially during periods when prey populations are low. Additionally, sugar dispensers on tomato plants can help manage mirid populations by curbing phytophagy, thus reducing plant damage while ensuring that predatory behaviors against pests remain effective [44]. Floral and extra floral nectar sources further contribute to an IPM strategy by enhancing the density and effectiveness of biological control agents, facilitating their ability to keep *T. absoluta* and other pests under control [45].

### 2.3. Shelter, Habitat Structure, and Environmental Stability

Structural elements within agricultural landscapes are pivotal in enhancing biocontrol agents’ survival, dispersal, and efficacy in targeting *T. absoluta*. Techniques such as intercropping, companion planting, and the establishment of ecological infrastructures, such as flower margins, provide critical shelter and create microhabitats that mitigate environmental stressors, including extreme climatic conditions and pesticide exposure. For instance, companion planting with *S. indicum* has been demonstrated to reduce plant damage associated with *N. tenuis*, thereby enhancing pest control in tomato crops while minimizing plant harm [33,51].

Crop architecture and adjacent natural habitats facilitate the colonization of predators and parasitoids, enabling their effective dispersion within agricultural systems. Such habitats supply nutritional resources, including pollen and nectar, alongside structural complexity, which collectively fulfill the ecological requirements of biocontrol agents. These integrations improve predator and parasitoid survival and bolster their performance within IPM frameworks [52,53]. Moreover, incorporating features such as natural vegetation strips and mulch layers enhances environmental stability by conserving soil moisture and providing protection against adverse conditions. These interventions contribute to the long-term sustainability of biological control programs by maintaining stable populations of natural enemies [54].

As summarized in Table 2, various habitat features play crucial roles in supporting biocontrol agents. For example, natural vegetation strips enhance predator persistence and dispersal, while artificial shelters provide critical refuges during adverse conditions. Similarly, mulch layers and perennial ground cover create favorable microhabitats, ensuring consistent support for predator establishment and reproduction. Shelter belts reduce wind speed and provide stable conditions for biocontrol agents, further reinforcing their effectiveness in pest management. These habitat features collectively improve the resilience and efficiency of biological control strategies.

Using banker plants represents another effective strategy, particularly in controlled environments such as greenhouses. Banker plants provide alternative prey or hosts, supporting the establishment and reproduction of natural enemies before pest infestations occur. This proactive approach enables predators to build their populations in advance, ensuring their readiness to suppress pests when infestations emerge [41]. This method has shown substantial efficacy with generalist predators, including mirid species, which benefit from structured habitats that simultaneously fulfill their shelter and nutritional needs.

Interactions between *T. absoluta* and other pest species within the same crop can further enhance the efficacy of biological control by promoting prey switching among generalist predators. This dynamic interaction contributes to pest suppression within IPM programs [7,55]. Strategic habitat modifications, including shelter plants and flower margins, create favorable conditions for natural enemies, reduce reliance on chemical pesticides, and support the implementation of sustainable pest management strategies in tomato cultivation.

**Table 2:** Influence of Shelter and Habitat Structure on the Performance and Stability of Biocontrol Agents in *T. absoluta* Integrated Pest Management.

Habitat Feature	Biocontrol Agent(s)	Observed Benefits	Description	Citation
Natural vegetation strips	*N. tenuis, M. pygmaeus*	Enhance persistence and dispersal of predators; provides continuous cover for natural enemies	Effective in reducing pest populations by maintaining predator stability in regions such as Northeast Spain and Southeast France	[41]
Artificial shelters	*N. artynes, Bracon nigricans*	Protect from adverse environmental factors	Offer refuge, especially during climatic extremes or pesticide application	[56]
Hedge rows	*N. tenuis*	Reduce pesticide drift and enhance biodiversity	Acts as a buffer zone, providing environmental stability and habitat diversity	[57]
Mulch layers	*M. pygmaeus*	Create a favorable microclimate and support predator establishment	Conserve soil moisture and improve local microhabitats	[58]
Perennial ground cover	*N. tutae*	Support continuous habitat for reproduction	Provide constant habitat for overwintering biocontrol agents	[33]
Shelter belts	*N. tenuis, N. artynes*	Reduce wind speed and maintain stability for biocontrol agents	It is essential for providing consistent shelter and stabilizing agent populations in open fields	[59]

## 3. Nutritional Ecology of Biocontrol Agents in Enhancing Biological Control Efficacy

The study of nutritional ecology in biocontrol agents, particularly parasitoids, has revealed critical insights into how various food resources enhance their pest control potential. As adults, most parasitoids and many predators rely on sugar-rich resources to meet their energy requirements, influencing longevity, fecundity, and overall fitness, which are vital for effective pest control. Historically, practitioners have sought to boost the nutritional state of these agents by introducing flowering plants and artificial sugars in agroecosystems [60,61]. In many ecosystems, honeydew also represents a primary carbohydrate source, offering an often-overlooked nutritional reservoir [62]. This section explores biocontrol agents’ sugar and protein requirements, the impacts of nectar quality and accessibility, and the importance of spatial and temporal availability of floral resources for sustaining biocontrol efficacy.

### 3.1. Sugar-Rich Resources and Their Impact on Biocontrol Agent Fitness

Sugar-rich resources, particularly floral nectar and honeydew, are crucial for supporting the fitness and effectiveness of biocontrol agents in agricultural systems. The absence of sugar intake significantly reduces the fecundity and lifespan of parasitoids, impairing their ability to control pest populations effectively [45]. Nectar, with sugar concentrations ranging from 20% to as high as 80%, typically consists of sucrose, glucose, and fructose, which serve as essential energy sources for parasitoids and predators, enabling them to forage across larger areas and interact with prey more efficiently [63,64]. These sugars are indispensable for maintaining the sustained activity of biocontrol agents, particularly in agroecosystems where pests such as *T. absoluta* threaten crop productivity. While nectar is often preferred because of its optimal sugar composition, honeydew, excreted by sap-sucking insects, provides an abundant and accessible carbohydrate source, particularly in large-scale agricultural settings; however, the nutritional quality of honeydew varies significantly, as some oligosaccharides it contains are not metabolizable by parasitoids [65,66]. Incorporating diverse sugar-rich resources in farming landscapes reduces biocontrol agents’ reliance on floral nectar, ensuring their prolonged activity and effectiveness in pest suppression.

Several studies have highlighted the role of flowering plants in enhancing the performance of natural enemies. For instance, the nectar of *Lobularia maritima* serves as a nutrient-rich food source for *N. tutae* and *Dolichogenidea gelechiidivoris* (Marsh) (Hymenoptera: Braconidae), significantly reducing the percentage of live *T. absoluta* larvae in tomato crops [39,67]. This finding underscores the importance of sugary foods in supporting the foraging behavior of parasitoids, which is essential for successfully controlling pests during the early stages of infestation; however, questions remain regarding the nutritional profiles of carbohydrates and proteins in *L. maritima* nectar and the volatiles it emits, which may attract both parasitic wasps and pests such as *T. absoluta*. Notably, volatiles from *L. maritima* have been shown to positively affect other parasitic wasps, such as Cotesia vestalis, enhancing their foraging behavior and parasitism rates [68]. Additionally, research has demonstrated that *C. vestalis* benefits from floral nutrients, maturing more eggs for parasitism and extending its reproductive capacity [18,68,69].

Beyond nectar, other floral resources, including pollen and extrafloral nectaries, contribute to the nutritional needs of biocontrol agents. For example, feeding on corn pollen has been found to significantly extend the longevity of *Trichogramma brassicae*, illustrating the value of non-nectar sugar sources [70]. Moreover, the presence of *Coriandrum sativum* (Apiales: Apiaceae) in agricultural landscapes has been linked to increased fertility and reproductive success in indigenous predators, emphasizing the importance of diverse plant assemblages with overlapping flowering periods [45,71]. Such habitats support biocontrol agents and enhance biodiversity and ecosystem services, creating more resilient agroecosystems. Floral nectar provides more than just sugars; it also delivers proteins, amino acids, and lipids, which are vital for fecundity and egg maturation in parasitoids [67]. Deliberate selection and integration of flowering plants, such as *L. maritima* and *C. sativum*, into cropping systems extend the availability of these resources, significantly boosting biocontrol efficacy. Collectively, these findings emphasize the need for integrating sugar-rich resources into pest management strategies to sustain and enhance the performance of biocontrol agents [18,65,68,72]. Sugar-rich resources, such as floral nectar and honeydew, are essential for enhancing the performance and sustainability of biological control agents, and their strategic incorporation into cropping systems can strengthen pest management while promoting ecosystem resilience.

### 3.2. Protein and Amino Acid Contributions to Reproduction and Longevity

Proteins and amino acids in nectar play a substantial role in biocontrol agents’ reproductive success and longevity. Specific amino acids, such as proline and serine, contribute to the development and fecundity of many insect predators and parasitoids [73]. Nectar amino acids are particularly valuable for synovigenic parasitoids, which produce eggs throughout their adult life and require continuous access to external nutritional sources for reproduction. Studies on parasitic wasps, for example, indicate that protein and amino acid-rich sources can significantly impact egg production, leading to higher pest suppression rates [74].

In addition to sugars and amino acids, certain parasitoid species also engage in “host-feeding” behavior, consuming hemolymph from their host insects. This behavior allows them to obtain proteins directly from their prey, supplementing the typically carbohydrate-based diet provided by nectar and honeydew [75]. Host hemolymph provides proteinaceous materials necessary for egg production, otherwise absent in nectar [76]. By offering both nectar-based amino acids and host-derived proteins, agroecosystems can improve parasitoid populations’ reproductive potential and sustainability, facilitating more consistent biocontrol efficacy.

### 3.3. Nectar Quality and Floral Accessibility as Drivers of Biocontrol Efficacy

**Figure 1:**
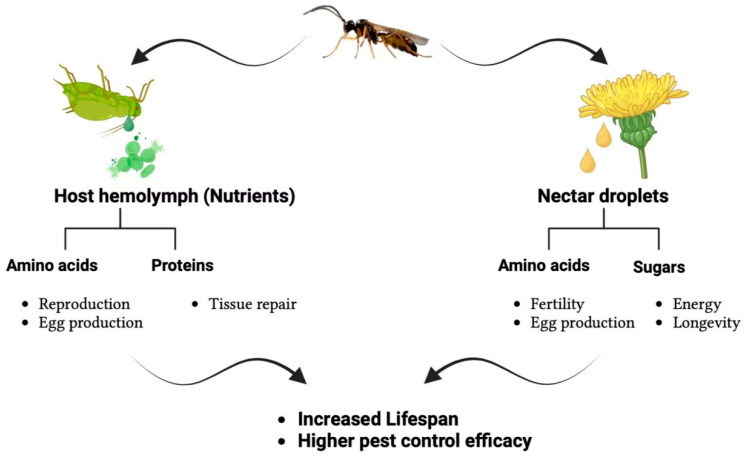
The figure illustrates predatory wasps’ dual nutrient acquisition strategies, emphasizing their dependence on host hemolymph and floral nectar. Host hemolymph, extracted from prey insects such as aphids, supplies amino acids that support reproduction and egg production and proteins essential for tissue repair. On the other hand, floral nectar provides amino acids that enhance fertility and egg production, along with sugars that offer energy and promote longevity. These nutrient sources synergistically improve the wasps’ lifespan and pest control efficacy, highlighting their critical role in ecological balance and integrated pest management.

Nectar quality and accessibility are critical determinants of biocontrol agent feeding behavior and efficacy. Nectar compositions are rich in sugars, amino acids, and bioactive compounds, which optimize feeding efficiency and provide essential energy and reproductive benefits for sustained pest suppression [77]. Predatory wasps, for instance, rely on nectar sugars for energy and amino acids to enhance fertility and egg production, with these nutrients playing a vital role in extending their lifespan and improving pest control activity (Figure 1). Studies show that sugar concentration directly influences the energy expenditure and activity levels of biocontrol agents, increasing their ability to locate, pursue, and control prey [78]. Flower morphology, including corolla depth and structure, further affects nectar accessibility. Flowers with shallow corollas are generally more accessible, allowing a broader range of biocontrol species to utilize the nectar, whereas deep corollas may restrict access to species with specialized mouthparts [79].

Research has demonstrated that even slight differences in floral architecture can impact feeding behaviors, with more accessible flowers leading to higher feeding rates and, consequently, improved pest control outcomes [80]. Additionally, nectar quality varies significantly among plant species. Certain plants produce nectar containing secondary metabolites, which may deter or enhance feeding by specific biocontrol agents, affecting agent selection and pest suppression capabilities. Selecting flowering plants with optimal nectar compositions can improve the nutritional intake of biocontrol agents, making them more effective within agricultural landscapes [63]; however, it is essential to note that while nectar contributes significantly to biocontrol agents’ energy and reproductive potential, its role is most effective when combined with alternative nutrient sources, such as host hemolymph. For example, hemolymph-derived proteins aid tissue repair, while amino acids support egg production and reproduction. This synergy highlights the need for a diverse habitat that supports both floral and prey availability to maximize the efficacy of biocontrol agents. Future research should explore the precise balance of these nutrient sources and how agricultural landscapes can be optimized to sustain these essential ecological interactions.

### 3.4. Temporal and Spatial Availability of Floral Resources in Supporting Biocontrol Populations

Temporal resource continuity is vital for the lifecycle requirements of biocontrol agents. Many arthropods have stage-specific or season-specific resource needs, such as parasitoid wasps that depend on caterpillars during their larval stages but shift to nectar as adults or predatory beetles that use herbaceous vegetation in summer but overwinter in wooded areas [81,82]. Recognizing these dynamics, the movement of natural enemies into crops and their spillover back to natural vegetation is essential for maintaining population stability and resource access [83,84]. Ensuring such continuity can promote early recruitment of natural enemies, a key factor for effective pest suppression populations, essential for managing pests such as *T. absoluta* within sustainable IPM frameworks. Floral resources provide critical support for biocontrol agents, particularly during periods of low pest density, ensuring their persistence and readiness for pest outbreaks. This approach is widely applicable across agroecosystems, but a persistent bias in research has focused predominantly on developed regions [85,86].

Strategic spatial and temporal arrangements of floral resources are indispensable for biocontrol efficacy. Flower strips adjacent to fields provide immediate access to nectar and shelter from environmental stressors and pesticides [87]. These strips and a diversity of flowering plants staggered across bloom times ensure year-round resource availability. For example, continuous flowering supports parasitoid survival and prevents population bottlenecks that could undermine pest control [88,89]. Field studies corroborate these benefits: Balzan and Moonen [90] found increased natural enemy abundance and pest suppression in tomato fields integrated with flowering plants, while [19] reported enhanced parasitoid efficacy against *T. absoluta* in similar setups. Moreover, these strategies can foster biodiversity, creating an agroecosystem that supports a broader range of beneficial organisms beyond biocontrol agents.

**Figure 2:**
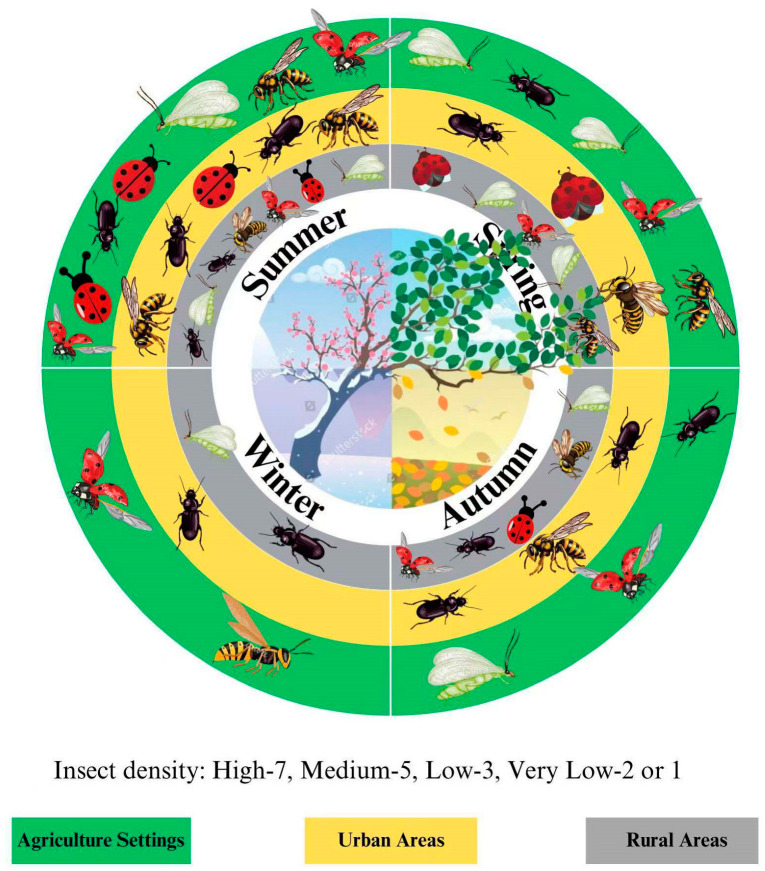
The circular diagram illustrates seasonal variations in insect density (High, Medium, Low, and Very Low) across three distinct settings: agricultural, urban, and rural areas. The inner sections depict seasonal changes affecting insect activity in spring, summer, autumn, and winter. Agricultural settings have the highest density of beneficial insects, such as lady beetles and parasitoids, supported by deliberate floral resource management. Urban areas maintain moderate densities, while rural regions show lower densities, reflecting limited floral resource availability. The visualization underscores the importance of floral resource planning in maintaining biocontrol agents’ populations year-round.

The availability of specific floral resources, such as *L. maritima*, demonstrates context-dependent effects on biocontrol agents. While it supports parasitic wasps such as *N. tutae* under low host density conditions, it provides negligible benefits in high host density scenarios [18,19]. These findings highlight the need for tailored resource integration strategies based on specific crop–pest–biocontrol contexts and emphasize the importance of further research on long-term effects across growing seasons. Additionally, variations in floral resource availability must be assessed to prevent disruption of ecosystem balance by non-target species. Through intentional floral management, agricultural settings provide the highest biocontrol agent densities, while urban and rural areas show moderate to low densities due to limited floral resources [91]. This underscores the need for spatial and temporal floral resource planning for effective pest suppression. Integrating floral diversity into agricultural landscapes ensures stable biocontrol populations, reduces reliance on synthetic pesticides, and enhances pest management sustainability [92]. By aligning floral resource availability with biocontrol agents’ life cycles and nutritional needs, agricultural systems can achieve dual benefits, supporting biocontrol populations and enhancing resilience against pests such as *T. absoluta*. The seasonal dynamics of biocontrol populations, as shown in Figure 2, emphasize the importance of strategic floral planning for optimal pest control.

## 4. Synergistic Effects of Floral Resources on Biocontrol Agent Dynamics

### 4.1. Behavioral Patterns and Floral Resource Utilization

Conservation biological control (CBC) strategies focus on enhancing the environment to sustain natural arthropod enemies and maximize their efficacy in pest management through habitat modifications that provide essential resources [93]. Floral resources, such as nectar and pollen, have been shown to positively influence the movement, foraging behavior, and predation efficiency of biocontrol agents, thereby contributing to improved pest suppression in agricultural landscapes. By providing sustenance, floral resources support the longevity and fecundity of predators and parasitoids, critical factors for sustained biocontrol activity [78]. In diversified agroecosystems, where multiple crop and non-crop plants create varied phonologies, natural enemies often face challenges due to limited shelter and food, which are less accessible in simplified, monoculture-dominated landscapes. Here, the introduction of flowering plants around or within crops can provide crucial non-host-derived nutrients, including nectar from flowers and extra floral nectarines, which enhance biocontrol agents’ ability to locate, subdue, and reproduce effectively in proximity to pest populations [94].

Floral volatiles are crucial in mediating the attraction of biocontrol agents such as parasitoids and predators. These compounds, including terpenes, phenylpropanoids, and fatty acid derivatives, act as chemical cues that guide natural enemies to their hosts or prey. For example, terpenes such as linalool and pinene and phenylpropanoids such as eugenol are known to attract parasitoids such as *N. artynes* [18]. Fatty acid derivatives such as (E)-2-hexenal, emitted by flowers such as *S. aemula*, also enhance the foraging efficiency of biocontrol agents [19]. The presence of these volatiles signals the availability of both floral resources and pest populations, improving pest control by guiding natural enemies to infested areas [1].

Chemical ecology further aids CBC by identifying plants that can attract beneficial insects, improving crop pest suppression. Traditionally, CBC studies have emphasized attraction via insect-produced kairomones and herbivore-induced plant volatiles (HIPVs), which increase host-seeking efficiency among natural enemies [95]; however, the role of floral odors in this context, while comparatively underexplored, is crucial, as these scents communicate flower identity and nectar quality, potentially guiding biocontrol agents to beneficial plants [96]. Integrating flowering plants with desirable chemical cues could enhance CBC efforts, aligning pest control with sustainable, ecology-based agricultural practices.

### 4.2. Influence of Floral Resources on Parasitoid-Host Interaction

**Figure 3:**
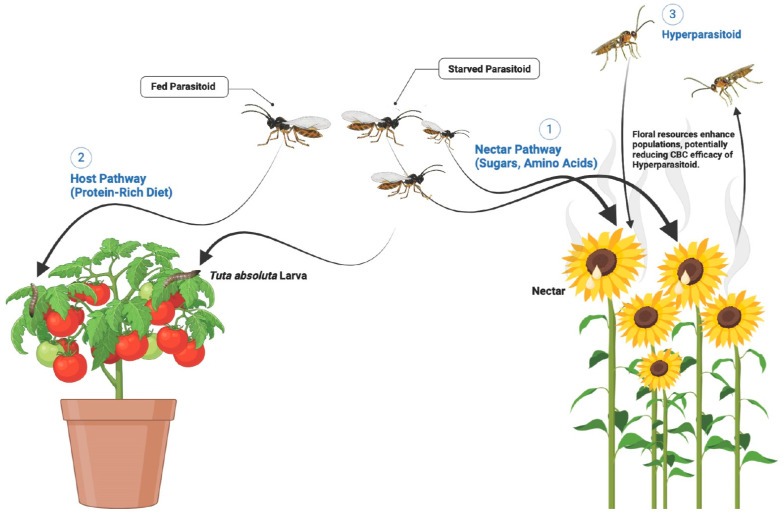
The figure illustrates the interaction between parasitoids, their prey (e.g., *T. absoluta* larvae), and the role of nectar in influencing biological control outcomes. 1. Nectar Pathway (Sugars, Amino Acids): Parasitoids that access nectar resources gain sugars for energy and amino acids for reproduction. 2. Host Pathway (Protein-Rich Diet): Parasitoids also derive proteins by parasitizing *T. absoluta* larvae, which supports their reproductive success and fitness. 3. Impact of Floral Resources on Hyperparasitoids: This highlights how nectar availability supports hyperparasitoid populations. At the same time, nectar resources are crucial for maintaining the primary parasitoid population.

Floral resources significantly shape parasitoid-host interactions by providing essential nutrients that enhance parasitoid longevity, reproductive success, and efficacy in *T. absoluta* biocontrol. Floral odors, which carry information about the flower’s identity and nectar quality, can attract pollinators and parasitoids seeking nutrition for sustained parasitism activity. Model simulations reveal that parasitoids are more advantageous in visiting beautiful flowers with limited nectar accessibility, as these yield greater longevity and parasitism success than readily accessible but less attractive flowers [97]. Beyond this direct attraction, microbes present in floral nectar can modify nectar’s chemical composition, including sugar and amino acid profiles, through microbial volatiles. This microbial influence can alter floral attractiveness and nutrient availability for parasitoids, complicating the interactions between flowering plants and biocontrol agents [98]. Nutritional pathways play a pivotal role in shaping the efficacy of parasitoid-based biological control systems. Parasitoids benefit from two primary nutrient sources: nectar, providing sugars for energy and amino acids for reproduction, and protein-rich hosts, such as *T. absoluta* larvae, which support reproductive success; however, while floral resources enhance the survival and efficiency of primary parasitoids, they can also bolster hyperparasitoid populations, potentially undermining CBC strategies by reducing the primary parasitoid’s pest suppression capabilities (Figure 3). These changes introduce a “hidden” factor in CBC, as microbes may indirectly shape how parasitoids utilize floral resources [77]. Further complicating matters, hyperparasitoids—natural enemies of parasitoids—may also be attracted to the same resources, potentially reducing the effectiveness of CBC by preying on primary parasitoids [99].

The search for food and hosts in parasitoids is closely linked to their nutritional state. For example, starved *C*. *vestalis* females preferred floral scents over vegetative plant parts, indicating that energy-deprived parasitoids prioritize feeding [100]. On the other hand, well-fed parasitoids focused more on host searching, underscoring how adequate nutrition from floral resources can balance foraging behavior to improve parasitism. In monoculture-dominated agriculture, a lack of flowering vegetation limits food availability, often leaving parasitoids starved, significantly reducing their efficacy as biocontrol agents [40]. Nectar’s value goes beyond sugars, as it contains essential amino acids, proteins, and organic acids necessary for ovigenesis, particularly in synovigenic parasitoids that rely on adult-acquired nutrients for reproduction [101]. Floral nectar supports longevity for *T. absoluta* parasitoids, such as *N. artynes*, while protein-rich diets, typically sourced from pest larvae, are crucial for sustained egg production [18,102]. Floral resources not only attract parasitoids but also support their foraging and reproductive activities, which are critical to the success of CBC. The selective management of floral resources is essential to mitigate risks associated with hyperparasitoid attraction and microbial alterations to nectar, thus ensuring optimal efficacy in *T. absoluta* IPM.

## 5. Case Studies on the Use of Floral Resources to Enhance Biocontrol of *T. absoluta*

While research on the impact of floral resources, specifically on *T. absoluta*, is still being conducted, several case studies provide evidence of the measurable benefits of using flowering plants to support biocontrol agents. Balzan and Wäckers [18] investigated the effects of selected flowering plants on the fitness and efficacy of *N. artynes*, a parasitoid of *T. absoluta*. Their findings revealed that floral nectar from *L. maritima* increased parasitoid lifespan by an average of 35%, while nectar from buckwheat (*Fagopyrum esculentum*) improved survival by 28%. Importantly, these benefits were not observed for *T. absoluta*, demonstrating the potential for targeted floral resources to strengthen biological control while avoiding unintended support for the pest.

Similarly, Kandori et al. [46] explored the use of *V. hybrida* and *S. aemula* as banker plants to sustain populations of *N. tenuis*, an effective predator of *T. absoluta*. These plants provided sugars, including fructose and glucose, which increased predator reproduction by 40% and survival by 50% in greenhouse environments. This approach reduced the dependency on alternative prey, making banker plants a practical solution for sustaining predator populations and improving pest control efficacy.

Arnó et al. [10] conducted a comparative evaluation of flowering plants to determine their suitability for supporting parasitoids of *T. absoluta*. They found that *L. maritima* and *Fagopyrum esculentum* significantly increased the longevity and egg load of *Necremnus tutae* and *B. nigricans* by 30–50% without extending the survival of *T. absoluta*. These results highlight the strategic value of these plants in IPM programs. In contrast, plants such as *Achillea millefolium*, *Calendula officinalis* (Asterales: Asteraceae), and *Sinapis alba* (Brassicales: Brassicaceae) provided mixed results, emphasizing the importance of plant selection based on specific biocontrol needs and environmental conditions.

## 6. Challenges and Future Directions

Integrating floral resources into IPM programs to control *T. absoluta* offers promising avenues for enhancing biocontrol efficacy, yet comes with significant challenges [103,104,105]. One primary challenge is achieving selective attraction, as floral resources ideally support beneficial species such as *N. artynes* and *N. tenuis* without unintentionally attracting other, less beneficial organisms or pests. [106]. Floral traits, such as specific volatile profiles, could be harnessed to ensure these resources appeal mainly to targeted biocontrol agents, thus minimizing the risk of attracting competitors or unintended species [107,108,109]. A related challenge is environmental variability, which can cause the efficacy of floral resources to fluctuate across different conditions [110]. Nectar quality, for example, can be highly influenced by climate, seasonality, and soil composition, affecting its role in supporting biocontrol agents [63,111]. This variability complicates the reliable application of IPM, as consistent pest control across diverse environments is necessary to ensure that the systems can be applied broadly and effectively [112]. The microorganisms in floral nectar add complexity to the utility of these resources. Nectar-inhabiting bacteria and fungi can alter the chemical makeup of nectar, sometimes changing its sugar concentration or amino acid profile, which can directly impact its attractiveness and nutritional value for biocontrol agents [113,114]. Such microbial modifications can significantly influence the survival, fecundity, and overall effectiveness of parasitoids or predators that rely on these nectar sources [115,116]. This potential for microbial impact reveals a gap in the current knowledge of IPM, suggesting further research to understand how these organisms influence biocontrol dynamics and whether they can be managed or leveraged to improve nectar quality [117,118].

Another critical factor is the temporal and spatial availability of floral resources, as inconsistent flowering periods or inadequate spatial arrangements can result in “resource gaps” that leave biocontrol agents without essential nutrients during key times in the pest control cycle [119]. Ensuring a continuous nectar supply throughout the growing season is crucial, as it helps maintain biocontrol agent populations through periods of low pest density, which would otherwise lead to population declines and reduce pest control efficacy when pest numbers rise [66]. Strategies such as staggered planting of various flowering species with different bloom times or positioning flower strips adjacent to crop fields are recommended to provide a steady flow of resources [120]. This targeted spatial and temporal deployment of resources not only sustains agent populations but can also provide accessible food sources and shelter from environmental stressors, thereby enhancing survival and performance [121]. Future research directions should prioritize the development of floral resource strips designed to selectively attract key biocontrol agents without benefiting pests such as *T. absoluta* or hyperparasitoids that prey on these beneficial insects [47,122]. By studying the floral traits that appeal specifically to natural enemies, such as unique volatile compounds or nectar profiles, researchers may create floral resource profiles tailored to maximize attraction to beneficial species while deterring others [123]. Such advancements could refine IPM strategies by providing targeted flower strips that effectively enhance pest control with fewer unintended consequences. Another promising area is exploring the role of nectar-associated microorganisms, as understanding how these microbes influence nectar chemistry could offer new ways to optimize floral resources for IPM [124]. Beneficial microbes might improve nectar quality, increasing biocontrol agents’ longevity and reproductive success; microbial volatiles could even act as chemical cues that enhance parasitoid attraction to appropriate flowering plants [77]. Future research could lead to microbial management strategies that further support biocontrol dynamics.

Optimizing temporal and spatial floral resource deployment through field studies would provide essential insights into the best practices for sustaining agent populations. Such studies could identify the most effective plant combinations for staggered flowering times, ensuring a continuous supply of nectar that bolsters agent presence across the growing season [125]. The strategic spatial placement of flower strips adjacent to or within crop fields can also retain biocontrol agents near pest populations, reducing the need for chemical inputs and allowing a steady, natural pest suppression response [126]. Additionally, such arrangements may help mitigate environmental stressors, such as pesticide drift, protecting biocontrol agents from harm [127]. Integrating banker plants with floral resources offers a complementary approach, where banker plants provide a steady source of prey or nectar for biocontrol agents, reducing the reliance on pests as food sources during off-peak pest periods. Banker plants such as *V. hybrida* and *S. aemula* have proven effective in supporting *N. tenuis* populations in greenhouse settings, thus reducing the need for chemical interventions [128].

Advances in chemical ecology could be pivotal in refining IPM systems further. Floral volatiles are specifically attractive to beneficial insects but unappealing to *T. absoluta* or hyperparasitoids, so they could be isolated and integrated into IPM strategies to strengthen biocontrol dynamics. Manipulating these volatiles could create flower strips that attract biocontrol agents and deter pests, adding a layer of pest management and reducing pest establishment near crop fields. This understanding of floral scents and preferences could foster selective attraction, ensuring that beneficial insects benefit while minimizing unintended attraction or pest facilitation risks.

The integration of floral resources into IPM systems represents a promising approach to enhance the biocontrol efficacy of *T. absoluta* by providing continuous support for beneficial agents; however, realizing the full potential of this approach requires addressing several complex challenges, including achieving selective attraction of natural enemies, adapting to variable environmental conditions, mitigating microbial influences on nectar, and optimizing the temporal and spatial availability of resources. Future research should focus on identifying floral species with the precise traits needed to attract and support biocontrol agents without benefiting pests or hyperparasitoids. Additionally, exploring the microbial ecology of nectar and its interactions with floral resources could further enhance the effectiveness of biocontrol agents.

## Figures and Tables

**Table 1:** Key Floral Resources Supporting Biocontrol Agents in the Management of *T. absoluta*. This table summarizes various flowering plants, their associated natural enemies, and specific benefits to biocontrol efficacy based on recent findings in integrated pest management (IPM) strategies.

Floral Species	Associated Biocontrol Agent(s)	Specific Benefits	Citation
*Lobularia maritima* (Sweet Alyssum)	*N. artynes*, *N. tenuis*	Increase longevity and fecundity; provides nectar and shelter for agents	[18]
*Fagopyrum esculentum* (Buckwheat)	*Necremnus tutae*, *Bracon nigricans*	Enhance parasitoid survival and egg load	[19]
*Verbena × hybrida*	*N. tenuis*	Supply essential sugars; supports reproduction in greenhouse settings	[46]
*Scaevola aemula*	*N. tenuis*	Provide fructose and glucose, enhancing predator survival	[46]
*Centaurea cyanus* (Cornflower)	*N. artynes*	Support parasitoid survival and foraging behavior	[47]
*Calendula officinalis* (Marigold)	*M. pygmaeus*	Promote predator population density; effective in tomato crop margins	[41]
*Achillea millefolium* (Yarrow)	*B. nigricans*, *N. tutae*	Enhance parasitoid survival; does not benefit *T. absoluta*	[10]
*Sinapis alba* (White Mustard)	*N. tenuis*	Provide pollen as a protein source; supports reproduction and fitness	[18]
*Borago officinalis* (Borage)	*M. pygmaeus*, *N. tenuis*	Increase predation rates; provides nectar with high nutritional value	[48]
*Cosmos bipinnatus* (Cosmos)	*N. artynes*	Boost parasitoid fecundity and longevity	[49]
*Phacelia tanacetifolia* (Lacy Phacelia)	*N. tenuis*, *M. pygmaeus*	Enhance foraging efficiency and lifespan	[24]
*Sesamum indicum* (Sesame)	*N. tenuis*	Reduce plant damage risk while enhancing pest control	[50]
*Vicia sativa* (Vetch)	*N. artynes*, *B. nigricans*	Provide necessary proteins for egg production	[8]
*Lantana camara*	*N. tenuis*	Increase longevity; provides rich nectar for biocontrol agents	[48]

## Data Availability

No new data were created or analyzed in this study. Data sharing is not applicable to this article.
